# The Relationship between Mushroom Intake and Cognitive Performance: An Epidemiological Study in the European Investigation of Cancer—Norfolk Cohort (EPIC-Norfolk)

**DOI:** 10.3390/nu16030353

**Published:** 2024-01-25

**Authors:** Sara Cha, Lynne Bell, Claire M. Williams

**Affiliations:** School of Psychology & Clinical Language Sciences, University of Reading, Whiteknights Road, Earley Gate, Reading RG6 6ES, UK; sara.cha@pgr.reading.ac.uk (S.C.); l.bell@reading.ac.uk (L.B.)

**Keywords:** epidemiological, older adults, mushroom intake, cognitive performance, global memory

## Abstract

The previous literature suggests that regular consumption of edible mushrooms may confer neuroprotective cognitive health benefits. To further investigate the possible association between mushrooms and brain function during ageing, data from a population-based study of diet and chronic disease (EPIC-Norfolk cohort) were analysed. Changes in mushroom intake were measured using a food frequency questionnaire at three health check (HC) points over an 18-year period, with participants categorised based on their consumption frequency. Cognitive performance was assessed at the final health check (3HC) via a battery of validated tests assessing a range of different cognitive domains. The findings revealed a significant reduction in mushroom intake over time, with 4.12% of the cohort giving up mushrooms after previously consuming them. At 3HC, mushroom consumers displayed better cognitive performance than non-consumers across multiple cognitive domains. This relationship was observed to be dose-dependent, with those consuming 1 or more portions per week showing the highest cognitive scores. These findings suggest that regular mushroom consumption may be beneficial for cognitive function during aging. Further randomised controlled trials will be needed to confirm any potential benefits of mushrooms on long-term cognitive health, alongside public health initiatives to promote mushroom consumption in this older-adult demographic.

## 1. Introduction

Ageing is typically associated with a plethora of behavioural and cognitive function changes, with reductions in global memory, executive function, mood, and daily living skills observed [1]. Scientific evidence has shown diet to be a significant modifiable factor in reducing age-related cognitive decline and the incidence of neurodegenerative disorders, with studies highlighting the beneficial effects of various food components on neurocognitive health. One important food group is culinary mushrooms, which are known to provide a source of protein, dietary fibre (primarily β glucan), water and fat-soluble vitamins, and phytochemicals such as ergothioneine and diterpenoids. These mushroom bioactives have previously been shown to act as anti-inflammatory agents, increasing the expression of antioxidant enzymes and promoting neurogenesis [2,3] and regulating synaptic signalling cascades and neurotransmitter release [4].

Currently, there are a number of epidemiological studies and randomised controlled trials (RCTs) in the literature that have examined the relationship between mushroom consumption and neurocognitive health and mental well-being. Evidence from epidemiology is convincing, with numerous studies demonstrating a positive association between consumption of a plant-rich diet (that includes mushrooms) and cognitive or mental health outcomes, e.g., [5,6,7,8,9]. However, these studies often do not specifically investigate the relationship between mushroom intake and cognitive or mental health outcomes, as any mushroom component of the data is inherently confounded by habitual intake of all types of fruits and vegetables in these studies. Several studies have attempted to further isolate the specific contribution of mushrooms to this relationship, revealing that those who regularly consume mushrooms exhibit better cognitive function and experience lower rates of mood disorders or neurodegenerative disease than those who never, or rarely eat mushrooms, e.g., [10,11,12,13,14,15,16]. However, this previous literature predominantly focuses on Japanese, Chinese, or Korean cohorts where a wide variety of different mushroom species are frequently consumed as part of a habitual Asian diet. Further studies that collect specific information on the varieties of mushrooms being consumed are recommended, so as to determine which may be most beneficial. It is also recommended that the current epidemiological literature be extended to Western populations to see whether the same relationships are evident. In addition, it is important to try and isolate the direct contribution of mushrooms in observed relationships with cognitive health, in order to not confuse mushroom benefits with contributions from other fruits and vegetables. RCTs are better-able to examine mushroom benefits alone. However, previous RCTs have again focused mainly (though not exclusively) on Asian populations and have predominantly investigated exotic species of mushrooms such as Lion’s Mane, e.g., [17,18,19,20] or Reishi mushrooms, e.g., [21], rather than common dietary mushrooms. These studies have also shown significant variability in the cognitive tasks used, the age and health status of the participants tested, and the doses administered, making it difficult to draw definitive conclusions. However, it appears that Lion’s Mane may offer some mood benefits in menopausal women [22] and obese middle-aged adults [23] and may confer both mood and neuroprotective benefits in older adults [17,19]. The literature has not yet sought to investigate the types of commonly consumed mushrooms, such as white mushrooms (button, cup, or flat), portobello, porcini, or oyster mushrooms, which may be responsible for many of the epidemiological findings.

The current study begins to address some of the gaps in the literature by looking at epidemiological data from a Western ageing cohort. Indeed, the European Prospective Investigation of Cancer (EPIC-Norfolk) is one of the few longitudinal cohort studies that has collected information on participants’ mushroom intake, as well as fruit and vegetable intake, alongside data from a concurrent, comprehensive neurocognitive battery [24,25]. The objectives of the present study were as follows: (1) to utilize the EPIC-Norfolk multicentre cohort data to evaluate whether habitual consumption of mushrooms changes over time; (2) to assess whether dietary mushroom intake is associated with cognitive performance; and (3) to assess whether any significant relationship between mushroom intake and cognitive performance may be explained (in full or in part) by general fruit and vegetable consumption. We hypothesize that participants from the EPIC-Norfolk cohort who report regular mushroom consumption will score higher on neurocognitive tests assessing executive function, as well as visuospatial and prospective memory, compared to those who report low or no mushroom consumption. We also predict that this relationship will remain apparent when accounting for fruit and vegetable consumption. The data from the EPIC-Norfolk cohort provide a valuable resource for investigating the relationship between habitual mushroom intake and brain function during ageing. The findings of such research can be used to support current public health recommendations on diet, including increasing awareness of the benefits of mushrooms.

## 2. Materials and Methods

### 2.1. Study Cohort and Eligibility Criteria

The European Prospective Investigation of Cancer-Norfolk (EPIC-Norfolk) is a multi-cohort prospective study that has recruited over 30,000 people aged 40–92 years old living in the Norfolk area of the UK. The main goal of the EPIC-Norfolk cohort was to examine relationships between dietary factors and disease risk or markers relating to ageing. Researchers collected information about participants’ lifestyle and genetic factors from interviews, questionnaires, and blood samples. From 1993, participants were enrolled in the study and attended follow-up health checks at the following time points: 1997–1998 (1HC), 1998–2000 (2HC), 2004–2011 (3HC), 2012–2016 (4HC), 2016–2018 (5HC) [26].

To examine any changes in participants’ mushroom intake across time (Objective 1), data from food frequency questionnaires were obtained from the first three health check points from 1997 to 2011, as the data from later health check points was not yet available [25]. To investigate the relationship between mushroom intake frequency and cognitive performance (Objective 2), only data obtained from 3HC were included, since it was the only health-check that gathered concurrent data from a neurocognitive battery. To account for fruit and vegetable intake in the relationship between mushroom intake and cognitive performance (Objective 3), a later release of 3HC data were drawn upon, which included daily estimates of fruit and vegetable intake based on FETA analysis of the EPIC 3HC data (FETA is an analytical tool that processes EPIC FFQ data and generates detailed food group intakes [27]). At all health checks, participants provided informed consent and the studies were conducted based on the principles of the Declaration of Helsinki and the Research Framework Governance for Health and Social Care.

### 2.2. Measurement of Mushroom Intake

Dietary intake was assessed via the semi-quantitative EPIC-Norfolk Food Frequency Questionnaire (FFQ) and aimed to record participants’ average intake for different food groups within the last year [27,28]. The FFQ required participants to rate their consumption of individual foods within the main categories of fruits, vegetables, pasta, bread, meat, fish, dairy products, sweets, sauces, and drinks. Participants were required to stipulate their intake frequency from nine options including never or less than once/month; 1–3 portion/month; once a week; 2–4 portions/week; 5–6 portion/week; 1 portion/day; 4–5 portions/day; more than 6 portions/day. The data for the analysis of participants’ average mushroom intake were derived from these categorical data by converting to ratio data and results were reported as portions per week using a previously published method [12].

### 2.3. Assessment of Cognitive Function

Health check 3HC included a series of previously validated cognitive tests as part of a cognitive battery (EPIC-COG; summarised in Appendix A). These tests were used to assess different domains of cognitive function including attention, executive function, processing speed, reading skills, visuospatial, working, and prospective memory. Completion of the EPIC-COG battery took approximately 3 h.

### 2.4. Assessment of Fruit and Vegetable Intake

Health check 3HC included estimates of daily fruit intake (g/day) and daily vegetable intake (g/day). However, as the vegetable data also included a contribution from participants’ habitual mushroom intake, these vegetable data were adjusted to exclude any mushroom component. In order to do this, the ratio mushroom data obtained above were further converted from portions/week to g/day assuming a mushroom portion size of 45 g as reported by EPIC. These mushroom values were then subtracted from the daily estimates of vegetable intake.

### 2.5. Statistical Analysis

Data were first screened to ensure that only participants with complete datasets were included in our analyses. For Objective 1, only the participants with information on their frequency of mushroom intake across all three health checks were included. For Objective 2, complete datasets for both frequency of mushroom intake and cognitive test scores were needed. Participants with extreme values in their cognitive test scores or in their self-reported mushroom intake values (identified using 3*IQR rule) were excluded from the final analysis.

For Objective 1, ratio data were used in a repeated ANOVA with Bonferroni-corrected pairwise comparisons to examine any changes in average weekly mushroom intake between health check time points. Categorical data were used in a Cochran’s Q analysis with pairwise McNemar tests comparing the number of mushroom consumers versus non-consumers across each of the three health check points. For the analysis of Objective 2, a multivariate analysis of covariance (MANCOVA) was used to examine any significant differences in cognitive performance across four mushroom intake categories (“never or less than 1 portion/month”, “1–3 portions/month”, 1 portion/week”, or “more than 1 portion/week”) for each of the cognitive domains while also accounting for demographic covariates including age, gender, BMI status, and physical activity status. Bonferroni corrections were applied to all post hoc comparisons. For the analysis of Objective 3, the same statistical procedure as Objective 2 was used, but with the addition of habitual fruit intake and habitual vegetable intake (excluding mushrooms) as covariates in the MANCOVA model.

## 3. Results

### 3.1. Cohort Characteristics (Objective 1)

Of the 8623 participants in the 1HC, 2HC, and 3HC data sets, 5091 (59.04%) provided information on their frequency of mushroom intake at all three time points. In this cohort, 57.79% were females.

### 3.2. Change in Mushroom Consumption over Time (Objective 1)

A repeated ANOVA with a Greenhouse–Geisser correction showed that estimated mean weekly mushroom intake significantly differed between the three HC points [F(1.95, 9899) = 21.49, *p* < 0.001]. Bonferroni-corrected pairwise comparisons revealed that the average weekly mushroom intake significantly reduced from 1.42 (SE 0.02) portions at 1HC to 1.34 (SE 0.02) portions at 2HC (*p* < 0.001) and significantly reduced again to 1.30 (SE 0.02) portions at 3HC (*p* = 0.036). Furthermore, Cochran’s Q test indicated significant differences in the relative proportion of mushroom consumers versus non-consumers across the three health check points [ [χ^2^(2)] = 67.24, *p* < 0.001]. Pairwise McNemar tests revealed a significant increase in the proportion of non-mushroom consumers relative to mushroom consumers between 1HC and 2HC (*p* < 0.001), and between 2HC and 3HC (*p* < 0.001). Frequency data are shown in Table 1. Between 1HC and 3HC, 210 (4.12%) participants stopped regularly consuming mushrooms.

### 3.3. Cohort Characteristics (Objective 2)

Of the 8623 participants in 3HC, 5418 (62.83%) provided information for their mushroom intake frequency and had eligible EPIC-COG battery test scores. Table 2 provides a summary of participants’ demographic characteristics and their mushroom intake.

Most participants were of white ethnic origin (99.72%) and were cognitively healthy with an average score of 13.43 on the short form mini mental state exam (SF-MMSE). Furthermore, over half of the cohort (64.71%) were overweight or obese. More than half of the cohort (65.36%) also reported having a moderately inactive or inactive physical activity status. In terms of their mushroom intake, 82.74% regularly consumed mushrooms.

### 3.4. Relationship between Mushroom Intake and Cognitive Score Measurements (Objective 2)

**Figure 1:**
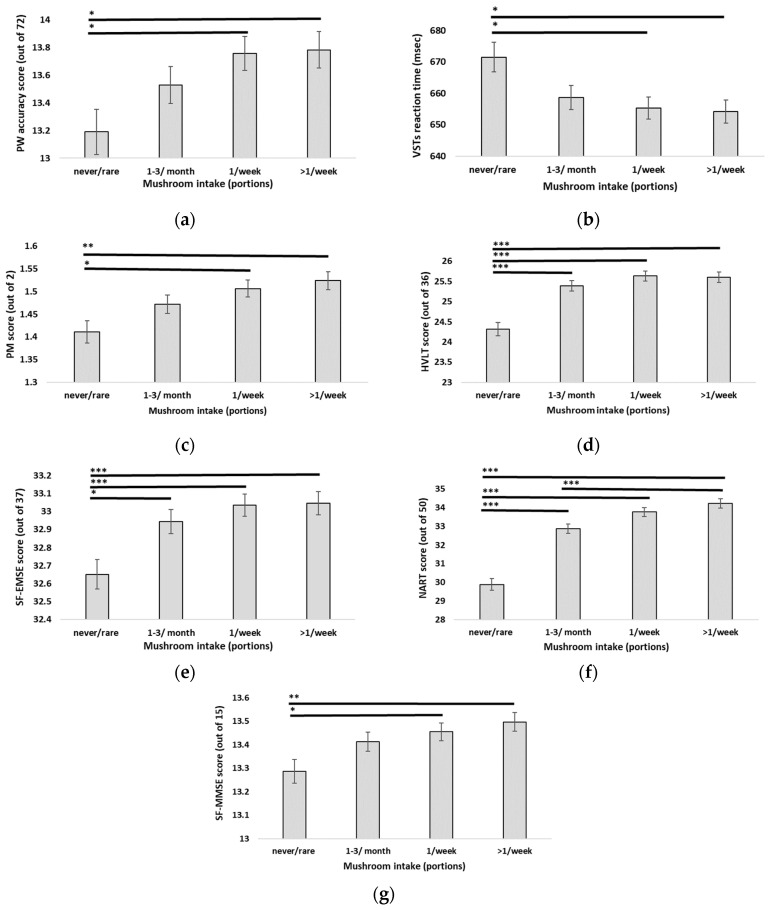
Cognitive test scores according to mushroom intake: (**a**) PW: pairwise test, (**b**) VST(s): Visual Sensitivity test (s: simple), (**c**) PM: prospective memory test, (**d**) HVLT: Hopkins Verbal Learning Task, (**e**) SF-EMSE: Extended Mental State Examination—short form, (**f**) NART: National Adult Reading Test, (**g**) SF-MMSE: Mini Mental State Examination—short form. Presented values are estimated marginal means with gender, age, BMI status, and physical activity status as covariates. Error bars represent standard error. * *p* < 0.05, ** *p* < 0.01, *** *p* < 0.001.

A MANCOVA was used to investigate differences in cognitive function across all cognitive tests between mushroom intake frequency groups: “never or less than 1 portion per month”, 1–3 portions per month”, “1 portion per week”, and “more than 1 portion per week”. Pillai’s trace revealed a significant relationship between mushroom intake and cognitive function [V = 0.03, F(27, 16,212) = 5.73, *p* < 0.001]. Separate univariate ANCOVAs on the individual cognitive measures revealed significant main effects of mushroom for all measures (*p* < 0.05), except for paired associate learning (CANTAB-PAL) (*p* = 0.063) and the complex visuospatial memory test (VSTc) (*p* = 0.285). All ANCOVA results for Objective 2 are presented in Supplementary Data Table S1. Significant cognitive outcomes are shown in Figure 1.

### 3.5. Accounting for Fruit and Vegetable Intake in the Relationship between Mushroom Intake and Cognitive Score Measurements (Objective 3)

Of the 5418 participants included in Objective 2, 5272 participants (97.21%) had daily fruit and vegetable intake data included in the 3HC FETA update. Of these, 2913 (55.25%) were female.

**Figure 2:**
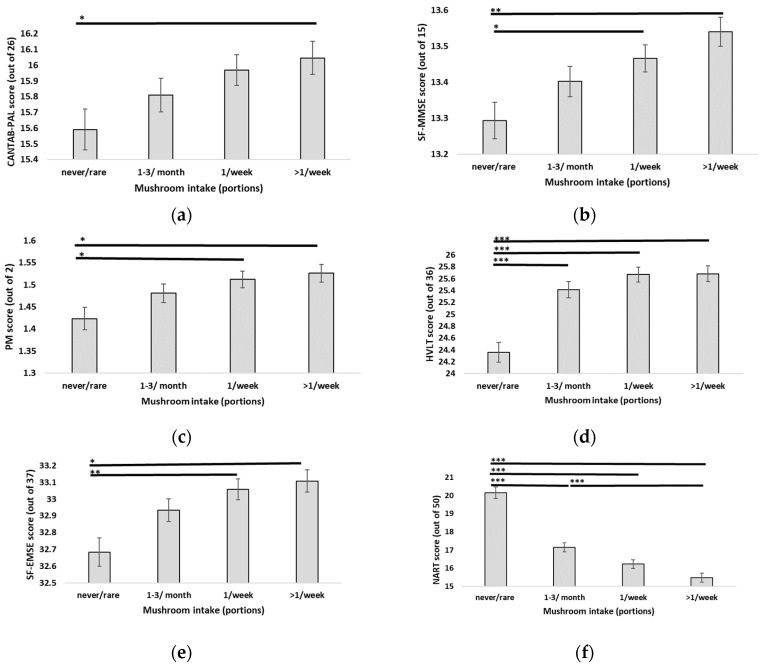
Cognitive test scores according to mushroom intake while accounting for fruit intake and vegetable intake (excluding mushrooms) as additional covariates: (**a**) CANTAB-PAL: paired associate learning test, (**b**) SF-MMSE: Mini Mental State Examination—short form, (**c**) PM: Prospective memory test, (**d**) HVLT: Hopkins Verbal Learning Task, (**e**) SF-EMSE: Extended Mental State Examination—short form, (**f**) NART: National Adult Reading Test. Presented values are estimated marginal means with gender, age, BMI status, physical activity status, fruit intake, and vegetable intake (excluding mushrooms) as covariates. Error bars represent standard error. * *p* < 0.05, ** *p* < 0.01, *** *p* < 0.001.

As in Objective 2, MANCOVA was used to investigate differences in cognitive function across all cognitive tests between mushroom intake frequency groups “never or less than 1 portion per month”, 1–3 portions per month”, “1 portion per week”, and “more than 1 portion per week”, this time with fruit intake and vegetable intake (adjusted to exclude mushroom intake) included as additional covariates in the analysis. While both additional covariates were found to be significant (*p* < 0.05), Pillai’s trace still revealed a significant relationship between mushroom intake and cognitive function [V = 0.03, F(27, 15,768) = 5.86, *p* < 0.001]. Separate univariate ANCOVAs on the individual cognitive measures revealed significant main effects of mushroom for all measures (*p* < 0.05), except for the visuospatial memory tasks: VSTs (*p* = 0.055) and VSTc (*p* = 0.523) (simple and complex versions of the task, respectively). However, although the pairwise task (PW) ANCOVA was statistically significant (*p* = 0.043), pairwise comparisons revealed no significant differences between individual mushroom-intake-frequency groups. Significant cognitive findings are shown in Figure 2. Interestingly, closer inspection of the additional covariates revealed that the fruit intake covariate was only found to be statistically significant for visuospatial memory (VSTs) and reading ability (NART) (both *p* < 0.05), whereas the vegetable intake covariate was significant for all cognitive tasks except for the pairwise task (PW), visuospatial memory (VSTc), and prospective memory (PM). Therefore fruits, vegetables excluding mushrooms, and mushrooms demonstrated some variation in their relationships with cognitive function across the different cognitive domains. All ANCOVA results for Objective 3 are presented in Supplementary Data Table S2.

## 4. Discussion

The aim of this epidemiological study was both to investigate consumption rates for mushrooms within an ageing cohort and to investigate the relationship between mushroom intake and cognitive function in a Western population. Findings showed that there was a significant reduction in participants’ mushroom intake frequency across the three EPIC health check points, with 4.12% of the cohort giving up mushrooms altogether. Importantly, mushroom intake was also found to be positively associated with cognitive performance across a range of cognitive skills, including prospective memory, executive function, and word recall, in this healthy ageing cohort. Covariates relevant to cognitive health, including gender, age, BMI status, and physical activity status were included in the primary analysis. Importantly, the findings still remained statistically significant after also accounting for fruit intake and vegetable intake (excluding mushrooms) as covariates in the model. However, it should be noted that additional covariates, such as education level, socioeconomic status (SES), or other measures of dietary health have not been included here. These factors were only examined during the HC1 health survey, not at the follow-up checkpoints, and data were not available at the time of the current analysis. However, while these factors are often associated with cognitive performance, other researchers have previously reported significant positive associations between mushroom intake and cognitive performance while also accounting for these additional factors [10,12,14]. Therefore, all findings should be accepted with the caveat that these other measures may also play a role in explaining (at least some of) the apparent relationship between mushroom intake and cognition. As a further caveat, given the cross-sectional nature of this study, it should also be noted that causal inference between mushroom intake and improved cognitive performance cannot be established and, indeed, the relationship may even be susceptible to reverse causality. RCTs will be required to establish causality and directionality.

Nevertheless, our findings are consistent with other epidemiological studies that have shown that consuming a healthy diet rich in vegetables (typically those including mushrooms such as a Japanese or Mediterranean style diet) is positively associated with beneficial cognitive outcomes, such as episodic memory and executive function, in both middle-aged and older populations [29,30]. Epidemiological studies specifically quantifying mushrooms in addition to general vegetable-rich diets have confirmed a clear association between mushroom consumption and better cognitive outcomes in older adults [11,31,32]. Importantly, this relationship has been shown to persist when accounting for additional confounding factors such as SES and dietary health [10,14]. In terms of dose, consumption of more than 12 g per day of fresh mushrooms (equivalent to 1 or more portions per week), shows a clear association with better cognitive scores in the domains of episodic memory, processing speed, and executive function [10,14]. This is consistent with the findings presented here that showed 1 or more portions per week to be associated with higher scores across a number of cognitive domains, including episodic memory, prospective memory, reading ability, executive function, and processing speed. Slightly higher consumption rates of more than 2 portions per week have also been associated with reduced odds of mild cognitive impairment [12]. Combined, these findings demonstrate that easily achievable intakes are associated with better cognitive outcomes, although it is important to clearly distinguish between portion sizes of fresh mushrooms compared with those of dried mushrooms, which are more concentrated and therefore are usually consumed in lower amounts. The findings from our study have been supported by similar findings from other epidemiological studies, but this type of methodological approach highlights some challenges in identifying the types of fresh or dried, wild or cultivated mushrooms that are habitually consumed, as food frequency questionnaires such as that used in EPIC-Norfolk do not go into a sufficient level of detail. It is important to gain a better understanding of the specific types of mushrooms consumed, particularly when comparing research across different cultures and when seeking to further investigate any causal effects of mushrooms on cognitive function using RCTs. Wild mushrooms typically have different nutrient profiles to cultivated mushrooms, so cognitive benefits may also vary between wild and cultivated types. Current RCT research has mainly focused on extracts from specialty mushrooms such as Lion’s Mane [33], and therefore experimental studies are likely not investigating the types of cultivated mushrooms commonly consumed as part of a habitual diet.

Potential mechanisms for bioactive effects of more commonly consumed mushrooms have been postulated from in vivo and in vitro animal research, with findings suggesting that these mushrooms are rich in fibre, anti-inflammatory, and antioxidant substances which are highly advantageous for cognitive health [34]. For example, oyster mushrooms rich in β-glucans, flavonoids, and ergothioneine have been shown to significantly enhance cognitive performance in rodents [11]. Similar effects have been observed for white button mushrooms [35], although this resulted from following higher dietary amounts due to relatively lower concentrations of the same bioactives in this mushroom type. Therefore, it seems feasible that culinary mushrooms such as these have the capacity to elicit cognitive benefits in humans when included as an integral part of the diet.

One of the additional objectives addressed here was to determine whether the observed significant relationship between mushroom intake and cognitive health would remain of significance when accounting for habitual fruit and vegetable intake. A criticism of this type of epidemiological research is often that other confounding factors such as general fruit and vegetable consumption may be responsible, either in part or in full, for the observed findings. However, here we have shown that mushroom benefits persist even when accounting for fruit and vegetable intake. An important point to note in the current analysis is that the level of fruit intake did not significantly account for variation in cognitive performance across a majority of the cognitive tasks investigated. Indeed, only reading level (NART) and simple visuospatial memory (VSTs) were significantly related to fruit intake. In contrast, vegetable intake was observed to significantly covary alongside most of the cognitive performance test scores recorded, with the exception of the pairwise task (PW), prospective memory task (PM), and complex visuospatial memory (VSTc). These findings suggest that vegetable interventions may have a greater impact on cognitive function during ageing than fruit interventions. Similar observations have been made by previous epidemiological research [36]. It is interesting to note that much of the nutritional psychology literature is taken up with fruit interventions, such as berries (including grapes), drupes (including plums and cherries), and citrus fruits, and very little experimental research has been carried out with vegetables. Therefore, future research should consider this untapped potential.

The epidemiological analysis presented here has considered the relationship between mushrooms and cognitive performance across a range of different cognitive domains, including memory and executive function. Throughout the healthy ageing process, it is expected that memory, mental speed, and agility may begin to decline, but a healthy diet rich in mushrooms and other vegetables may mitigate some of this natural decline. Importantly, there is also some evidence from previous epidemiological research that mushroom consumption may be associated with a lower risk of developing neurodegenerative diseases such as Alzheimer’s or other forms of dementia during ageing [31,37]. In healthcare, there is a clear distinction drawn between healthy ageing and the development of pathological diseases in old age, but it appears that mushrooms may convey benefits to both healthy and unhealthy ageing. In addition to the observed benefits to age-related cognitive performance, there is also evidence to suggest that mushroom consumption may be associated with better mood and mental health. In particular, studies have found a clear association between higher rates of mushroom consumption and lower rates of depression throughout the lifespan, ranging from young adulthood through to older age [13,15,38,39]. Therefore, mushroom benefits may be extended to people of all ages. The EPIC-Norfolk dataset does not record diagnoses of neurodegenerative disease or depression, so it was not possible to incorporate these outcomes in the current study. However, it is recommended that future nutrition studies consider these important aspects of wider cognitive health.

As discussed, several epidemiological studies have examined similar cross-sectional relationships between mushroom intake and cognitive function and aspects of mental health such as depression. However, this study is the first to also consider changes in mushroom consumption patterns during the transition from middle-age to old-age. Having identified that mushrooms may be beneficial for cognitive function, it is important to also understand how frequently they are currently consumed. Here, we have shown a decline in mushroom consumption in the EPIC cohort over time. While this might in part be explained by the lower nutrition requirements of older adults, who may simply be eating less of everything as they get older, it is important to note that a percentage of the cohort gave up mushrooms altogether. A further reason for the reduction in mushroom consumption might be attributed to health problems that develop during aging such as uric acid elevation (hyperuricemia) and the perception that all purine-rich foods, including mushrooms, should be avoided. However, recent research has demonstrated that mushroom consumption was actually associated with a lower risk of incident hyperuricemia in a middle-aged and older cohort sample [40]. Investigation of the reasons for the apparent reduction in mushroom consumption in the EPIC cohort would require further qualitative research, which was outside of the scope of this study, but future research should explore this further. Nevertheless, from a public health perspective, our findings may be helpful in raising awareness about recommended levels of mushroom intake and their potential cognitive health benefits.

## 5. Conclusions

The novel findings of this epidemiological study warrant further investigation but can be used to raise public health awareness about the potential benefits of mushrooms on cognitive function during ageing. The findings also highlight the importance of the relationship between vegetables and cognitive health during ageing, and in addition to further research into mushrooms, it is recommended that nutrition intervention research targets other vegetable interventions that may be similarly beneficial.

## Figures and Tables

**Table 1:** Average mushroom intake and frequency of non-consumers versus consumers across EPIC health check points.

N = 5091	1HC	2HC	3HC
Weekly portions (mean (SE))	1.42 (0.02)	1.34 (0.02)	1.30 (0.02)
Non-consumers	756 (14.85%)	858 (16.85%)	966 (18.97%)
Consumers	4335 (85.15%)	4233 (83.15%)	4125 (81.03%)

**Table 2:** Demographic characteristics of the EPIC-Norfolk 3HC cohort.

Characteristic	N = 5418
**Sex**	
Females	2995 (55.28%)
Males	2423 (44.72%)
**Age**	
<55 y old	145 (2.68%)
55–64 y old	1961 (36.19%)
65–74 y old	2213 (40.84%)
75–84 y old	1030 (19.01%)
>85 y old	69 (1.27%)
**Body Mass Index (BMI)**	
Underweight (<18.5)	39 (0.72%)
Normal weight (18.5–24.9)	1873 (34.57%)
Overweight (25.0–29.9)	2500 (46.14%)
Obesity class I (30.0–34.9)	781 (14.41%)
Obesity class II 35.0–39.9)	178 (3.29%)
Obesity class III (≥40.0)	47 (0.87%)
**Physical activity status**	
Active	880 (16.24%)
Moderate active	997 (18.40%)
Moderate inactive	1607 (29.66%)
Inactive	1934 (35.70%)
**Ethnic origin**	
White	5403 (99.72%)
Non-white/Other	15 (0.28%)
**Mushroom frequency intake**	
Never/rare	935 (17.26%)
1–3 portions/month	1381 (25.49%)
1 portion/week	1642 (30.31%)
2–4 portions/week	1287 (23.75%)
5–6 portions/week	137 (2.53%)
1 portion/day	36 (0.66%)

## Data Availability

Restrictions apply to the availability of these data. Data were obtained from the EPIC-Norfolk Study (https://www.epic-norfolk.org.uk/for-researchers/data-sharing/data-requests/ accessed on 4 December 2023).

## Supplementary Materials

The following supporting information can be downloaded at: https://www.mdpi.com/article/10.3390/nu16030353/s1, Table S1: Mean cognitive scores (estimated marginal means (SE) for each cognitive test based on mushroom intake frequency, n = 5418, with age, gender, physical activity status, and BMI status included as covariates; Table S2: Mean cognitive scores (estimated marginal means (SE) for each cognitive test based on mushroom intake frequency, n = 5272, with age, gender, physical activity status, BMI status, daily fruit intake, and daily vegetable intake (excluding daily mushroom intake) included as covariates.

## Appendix A. Description of the EPIC-COG Battery Tests Employed in the EPIC-Norfolk 3HC

**Cognitive Test**	**Domain**	**Description**	**Outcome Measure**
Extended Mental State Exam-Short Form (SF-EMSE)	Global memory (attention, abstract reasoning, and retrospective memory)	Items assessing higher function skills.	Score (0–37)
Hopkins Verbal Learning Task (HVLT)	Episodic and recognition memory	Word recall task	Words identified/3 trials (0–36)
National Adult Reading Test (NART)	Intelligent Quotient and reading skills	Word pronunciation task	Score (0–50)
Cambridge Neuropsychological Test Automated Battery-Paired Associate Learning (CANTAB-PAL)	Episodic memory and learning skills	Six white boxes presented on a touch screen, displaying 8 different visual patterns	Score (0–26)
Pairwise Test (PW)	Executive function, attention and processing speed	Visual scanning and crossing of the 72 P and W letters	Number of letters identified/min (0–72)
Time and event-based test (PM)	Prospective memory	Explicit instructions about tasks at a specific point	Success (100%)/Partial success (50%)/Failure (0%)
Mini-Mental State Exam—Short Form (SF-MMSE)	Memory screening tool (orientation, attention, recall and visuospatial ability)	Items assessing cognitive skills	Score (0–15)
