# Supplementary material for: The Relationship between Mushroom Intake and Cognitive Performance: An Epidemiological Study in the European Investigation of Cancer—Norfolk Cohort (EPIC-Norfolk)
Source: Nutrients. 2024 Jan 25;16(3):353. doi: 10.3390/nu16030353 (PMC10857520; doi:10.3390/nu16030353)
Supplement: Supplementary file 1 [file nutrients-16-00353-s001.zip › nutrients-2777962-supplementary.pdf]

Mushroom frequency intake	Cognitive test								
	**SF-EMSE**	**SF-MMSE**	**HVLT**	**NART**	**CANTAB-PAL**	**PW**	**PM**	**VSTs**	**VSTc**
**1. Never/rare intake (n=935)**	**32.65 (0.08)[2,3,4]**	**13.29 (0.05)[3,4]**	**24.32 (0.16)[2,3,4]**	**29.88 (0.31)[2,3,4]**	**15.56 (0.13)**	**13.19 (0.16)[3,4]**	**1.41 (0.03)[3,4]**	**672 (4.68)[3,4]**	**2197 (12.52)**
**2. 1-3 portions/month (n=1381)**	**32.94 (0.07)[1]**	**13.41 (0.04)**	**25.39 (0.13)[1]**	**32.87 (0.26)[1,4]**	**15.81 (0.11)**	**13.53 (0.13)**	**1.47 (0.02)**	**659 (3.84)**	**2176 (10.26)**
**3. 1 portion/week (n=1642)**	**33.04 (0.06)[1]**	**13.46 (0.04)[1]**	**25.63 (0.12)[1]**	**33.75 (0.24)[1]**	**15.97 (0.10)**	**13.76 (0.12)[1]**	**1.51 (0.02)[1]**	**655 (3.52)[1]**	**2186 (9.41)**
**4. >1 portion/week (n=1460)**	**33.05 (0.07)[1]**	**13.50 (0.04)[1]**	**25.60 (0.13)[1]**	**34.22 (0.25)[1,2]**	**15.93 (0.10)**	**13.78 (0.13)[1]**	**1.52 (0.02)[1]**	**654 (3.73)[1]**	**2168 (9.99)**
**Mushroom ANCOVA p value**	**0.001***	**0.010***	**<0.001***	**<0.001***	**0.063**	**0.019***	**0.002***	**0.020***	**0.285**

Table S1. Mean cognitive scores (estimated marginal means (SE) for each cognitive test based on mushroom intake frequency, n=5418, with age, gender, physical activity status, and BMI status included as covariates. [1,2,3,4] indicates significant Bonferroni-corrected pairwise comparison with the corresponding mushroom frequency intake group (p<0.05). * indicates significant p value (p<0.05). Abbreviations used: SF-EMSE: Extended Mental State Examination-short-form, HVLT: Hopkins Verbal Learning Task, NART: National Adult Reading Test, CANTAB-PAL: Cambridge Neuropsychological Test Automated Battery-Paired Associate Learning test, PM: Prospective memory test, PW: Pairwise test, VST(c,s): Visual Sensitivity test (c: complex, s: simple).

Mushroom frequency intake	Cognitive test								
	**SF-EMSE**	**SF-MMSE**	**HVLT**	**NART**	**CANTAB-PAL**	**PW**	**PM**	**VSTs**	**VSTc**
**1. Never/rare intake (n=897)**	**32.68 (0.08)[3,4]**	**13.29 (0.05)[3,4]**	**24.36 (0.17)[2,3,4]**	**20.15 (0.32)[2,3,4]**	**15.59 (0.13)[4]**	**13.30 (0.17)**	**1.42 (0.03)[3,4]**	**668 (4.73)**	**2190 (12.85)**
**2. 1-3 portions/month (n=1349)**	**32.93 (0.07)**	**13.40 (0.04)**	**25.41 (0.14)[1]**	**17.15 (0.26)[1,4]**	**15.81 (0.11)**	**13.56 (0.14)**	**1.48 (0.02)**	**660 (3.83)**	**2174 (10.40)**
**3. 1 portion/week (n=1602)**	**33.06 (0.06)[1]**	**13.47 (0.04)[1]**	**25.67 (0.12)[1]**	**16.23 (0.24)[1]**	**15.97 (0.10)**	**13.82 (0.12)**	**1.51 (0.02)[1]**	**655 (3.50)**	**2185 (9.50)**
**4. >1 portion/week (n=1424)**	**33.11 (0.07)[1]**	**13.54 (0.04)[1]**	**25.68 (0.13)[1]**	**15.49 (0.26)[1,2]**	**16.05 (0.11)[1]**	**13.84 (0.13)**	**1.53 (0.02)[1]**	**652 (3.79)**	**2169 (10.28)**
**Mushroom ANCOVA p value**	**0.001***	**0.002***	**<0.001***	**<0.001***	**0.040***	**0.043***	**0.010***	**0.055**	**0.523**
**Fruit covariate p value**	**0.072**	**0.385**	**0.644**	**0.003***	**0.528**	**0.617**	**0.284**	**0.013***	**0.330**
**Vegetable (excl. mushroom) covariate p value**	**0.001***	**0.001***	**0.035***	**<0.001***	**0.004***	**0.350**	**0.511**	**0.005***	**0.886**

Table S2. Mean cognitive scores (estimated marginal means (SE) for each cognitive test based on mushroom intake frequency, n=5272, with age, gender, physical activity status, BMI status, daily fruit intake, and daily vegetable intake (excluding daily mushroom intake) included as covariates. Covariate significance is included for fruit intake and vegetable intake. [1,2,3,4] indicates significant Bonferroni-corrected pairwise comparison with the corresponding mushroom frequency intake group (p<0.05). * indicates significant p value (p<0.05). Abbreviations used: SF-EMSE: Extended Mental State Examination-short-form, HVLT: Hopkins Verbal Learning Task, NART: National Adult Reading Test, CANTAB-PAL: Cambridge Neuropsychological Test Automated Battery-Paired Associate Learning test, PM: Prospective memory test, PW: Pairwise test, VST(c,s): Visual Sensitivity test (c: complex, s: simple).
